# Generation Mechanism and Prediction Model for Low Frequency Noise Induced by Energy Dissipating Submerged Jets during Flood Discharge from a High Dam

**DOI:** 10.3390/ijerph13060594

**Published:** 2016-06-15

**Authors:** Jijian Lian, Wenjiao Zhang, Qizhong Guo, Fang Liu

**Affiliations:** 1 State Key Laboratory of Hydraulic Engineering Simulation and Safety, Tianjin University, 92 Weijin Road, Nankai District, Tianjin 300072, China; zhangwenjiao2006@tju.edu.cn (W.Z.); fangliu@tju.edu.cn (F.L.); 2 Department of Civil and Environmental Engineering, Rutgers, The State University of New Jersey, Piscataway, NJ 08854, USA; qguo@rci.rutgers.edu

**Keywords:** LFN, high dam flood discharge, energy dissipation by submerged jets, vorticity fluctuation, prediction model

## Abstract

As flood water is discharged from a high dam, low frequency (*i.e*., lower than 10 Hz) noise (LFN) associated with air pulsation is generated and propagated in the surrounding areas, causing environmental problems such as vibrations of windows and doors and discomfort of residents and construction workers. To study the generation mechanisms and key influencing factors of LFN induced by energy dissipation through submerged jets at a high dam, detailed prototype observations and analyses of LFN are conducted. The discharge flow field is simulated using a gas-liquid turbulent flow model, and the vorticity fluctuation characteristics are then analyzed. The mathematical model for the LFN intensity is developed based on vortex sound theory and a turbulent flow model, verified by prototype observations. The model results reveal that the vorticity fluctuation in strong shear layers around the high-velocity submerged jets is highly correlated with the on-site LFN, and the strong shear layers are the main regions of acoustic source for the LFN. In addition, the predicted and observed magnitudes of LFN intensity agree quite well. This is the first time that the LFN intensity has been shown to be able to be predicted quantitatively.

## 1. Introduction

Noise pollution is a serious environmental problem to human beings, which has further been shown to be associated with reduced quality of life and wellbeing [1,2]. At present, most of the academic literature is related to high frequency noise sources from traffic, industry and human activities [3,4,5], while low frequency noise (LFN) is also considered annoying for humans [6]. The LFN found in living environments is mainly emitted from many artificial sources such as road vehicles, aircraft, and air movement machinery including wind turbines, compressors, and ventilation [7,8], and it is claimed that exposure to LFN has a negative impact on humans’ physiological and psychological health. The physiological problems include headaches, hormone changes, dizziness or vertigo, tinnitus and the sensation of aural pain or pressure, and the psychological impact can cause sleep disturbance, dysphoria, difficulty concentrating, irritability and fatigue [9,10].

In the field of hydraulic engineering in China, the majority of high dams were constructed in recent decades with the features of high water head, large flow capacity, a deep narrow valley and large flood discharge power. Issues such as energy dissipation and scour protection, vibration control of a hydraulic structure, vapor atomization protection, aeration for cavitation protection, and security warnings associated with high dam flood discharge have attracted significant attention and led to abundant technological achievements [11,12,13,14]. Some environmental problems from LFN have also recently been found around some hydropower stations during the flood discharge period. For instance, when the Xiangjiaba Hydropower Station (Zhaotong, at the border of Sichuan and Yunnan provinces, China) began to release flow through the dam orifices at a high water level on 10 October 2012, the roller shutter doors of some shops and the windows and doors of residential buildings experienced sustained vibration in downstream Shuifu County. The dominant frequencies of the LFN observed on-site were approximately 0–2 Hz. In a recent study [15], the impact of ground vibration induced by the flood discharge of Xiangjiaba on on-site roller shutter doors was eliminated by means of a vibration response analysis, and it was deduced that the doors shaking resulted from the resonant interaction between the flow-induced LFN and the door structures. Moreover, the windows of buildings located at the left abutment of the Ertan Hydropower Station (Panzhihua, Sichuan Province, China) oscillated noticeably when the flood was released. The windows and doors of residential buildings in a downstream village about 700–1500 m from the Huangjinping Hydropower Station (Kangding, Sichuan Province, China) experienced sustained vibrations when the flood was released. The LFN observed during the flood discharge period of both the Jinping Hydropower Station (Liangshan Yi Autonomous Prefecture, Sichuan Province, China) and Xiluodu Hydropower Station (Zhaotong, at the border of Sichuan and Yunnan provinces, China) had sound pressure levels (SPL) approximately 20–50 dB higher than the background noise, and the dominant LFN frequencies were between 0.5 Hz and 1.5 Hz. It is found that the inaudible LFN observed around these hydropower stations causes audible secondary noise from surrounding buildings. In Japan, there are some criteria specific to the vibration and rattle attributable to the effects of LFN from stationary sources in worksites, shops and neighborhood residences [16]. The lowest noise frequency listed in the criteria is 5 Hz with its reference SPL limit of 70 dB. For the flow-induced LFN, the LFN’s effects are extended to a lower frequency of about 0–2 Hz.

Japanese scholars have conducted some research on flow-induced LFN. As Japan is a densely populated country and many hydropower stations are close to residents’ living quarters, the problems of LFN were noted earlier in Japan. Most of the research focused on the waterfalls formed by the discharge flow through the weir or dam’s floodgate. The oscillation of such waterfalls can cause LFN, which has an adverse impact on surrounding buildings and inhabitants [17,18,19]. In addition, since LFN is a low frequency wave, it has a low attenuation rate in air [17,20,21], which is difficult to control, and thus can spread over long distances, even tens of kilometers, to resonate with buildings. Among the previous studies, Nakamura [17] reported the phenomenon of LFN induced by flood discharge as early as 1978. Takebayashi [18] and Nakagawa [19] carried out systematic research on the mechanisms of self-excited oscillation of cavity-waterfall systems and the characteristics of the induced LFN based on site observations. Some control measures, such as placing deflectors, were put forward and applied effectively in the research. Ochiai *et al*. [22] studied various influencing factors of the SPL of the noise from windows and doors induced by LFN by a series of model tests. Saitoh *et al*. [23] studied the sound sources of the hydraulic jump through hydraulic model experiments on the hydraulic jump and cylinder nozzle, and they found that the noise energy at a high frequency of 500–600 Hz induced by the hydraulic jump was derived from bubble cloud oscillations. These research results indicate that LFN induced by flood discharge can cause environmental problems within large areas, and the generation of LFN closely correlates with the discharge flow regime. The problems of LFN can be controlled and reduced by adjusting the flow regime. The previous studies mainly focus on LFN induced by a relatively continuous waterfall of some water projects with low water head, small flow rate and a simple flow regime. However, for water projects that dissipate energy through multi-horizontal submerged jets, LNF energy can still be observed on site although a waterfall does not exist during flood discharge. Therefore, there must be some differences in the generation mechanisms of LFN between energy dissipation by the submerged jet of high dam flood discharge and the oscillation of the waterfall. An extensive literature search has not found any research reports on LFN induced by energy dissipation through the submerged jets of a high dam.

The objective of this study was to identify the mechanisms and key influencing factors of LFN induced by energy dissipation through the submerged jets of a high dam. First, in light of the prototype observation results of LFN, the on-site spatial distribution and propagation laws of the induced LFN along the downstream are analyzed, and the correlation between LFN and the discharge flow regime is discussed. Next, the vortex sound model and numerical turbulent flow model for the flood discharge and energy dissipation of the high dam are presented as the theoretical bases. Then, the flow field’s distribution characteristics and effective regions of acoustic source for the LFN are studied and identified, according to the numerical simulation results of the gas-liquid turbulent flow model of flood discharge. The mathematical prediction model of the LFN intensity induced by the submerged jets is established based on the vortex sound model and turbulent flow model, and the prediction model is verified by prototype observation results. Finally, the findings are summarized, and the conclusions of this study are drawn.

## 2. Materials and Methods

### 2.1. Prototype Observation

#### 2.1.1. Prototype

**Figure 1:**
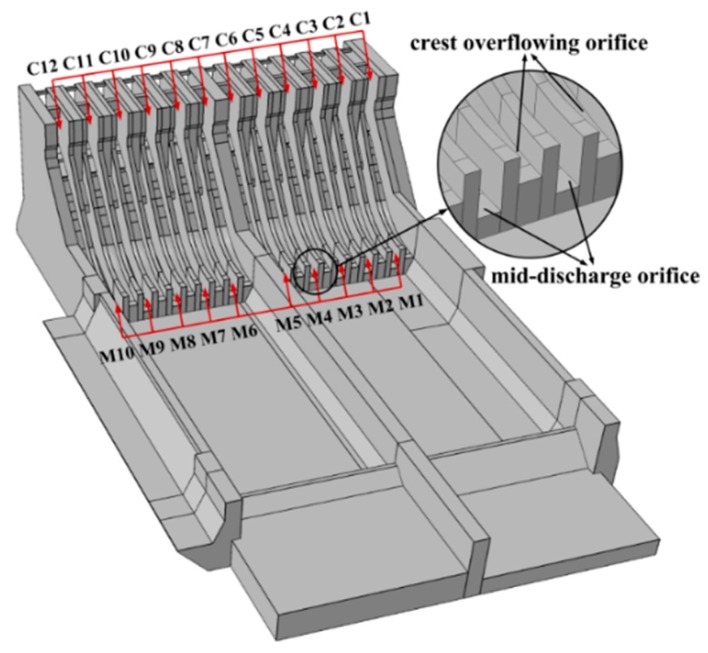
Layout of the water release structures.

The Xiangjiaba Hydropower Station is the third largest hydropower station in China and the sixth largest in the world. It is the last cascade on the Jinsha River and is adjacent to Shuifu County of Yunnan Province downstream, with the nearest distance being about 0.5 km [24]. The dam is 162 m in height, with a reservoir capacity of 5 × 10^9^ m^3^ and planned flood discharge rate of 41,200 m^3^/s. The total installed capacity of the station is 6400 MW. The station consists of water-retaining structures, flood-releasing and sediment-flushing structures, a water diversion and power generation system, navigation structures, and irrigation water intakes. Because of the large scale of the flood discharge and energy dissipation and the requirement of the flood discharge and sediment flushing of the hydropower station, the water release structure was chosen as a gravity spillway dam, composed of 12 crest overflowing orifices and 10 mid-discharge orifices, as shown in Figure 1. However, after comparing a range of orifice layout schemes through a hydraulic model test, it was determined that the water release structure had to dissipate energy by the submerged jets and that the 12 crest overflowing orifices and the 10 mid-discharge orifices ought to be alternately arranged for the sake of balancing the unit width discharge, facilitating flood discharge and sediment flushing, decreasing the sluice gate’s size and reducing the influence of the discharge atomization on the surrounding town and enterprise [25]. There are two symmetrical energy dissipation areas separated by the middle guide wall.

#### 2.1.2. Observation System and Conditions

Since 2012, Tianjin University has organized prototype observations of LFN many times during the flood period of Xiangjiaba. The observation equipment of LFN on site consists of an infrasound microphone, a multi-channel digitizer and a computer. The infrasound microphone, which was fixed above the ground a little over 2 m, has a frequency range of 0.1–500 Hz and a sensitivity of 107 mv/Pa. The multi-channel digitizer connects the microphone with the computer, which supplies power to the microphone and saves data in high speed. The digitizer has a built-in GPS, so the equipment can work well in the open air. With regard to the uncertainties in the observed data from the self-noise of the microphone and digitizer, the errors from the digitizer are small sufficiently to be neglected, and the maximum error from the microphone is below 2%, which corresponds to ±1 dB.

**Figure 2:**
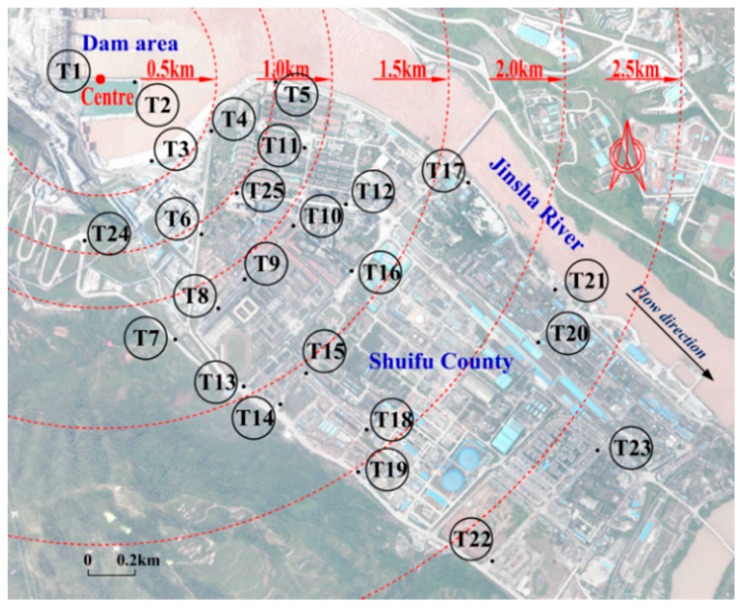
LFN observation point arrangement.

The observation range covered the dam area, construction area and urban area of Shuifu, placing 25 observation points in total (T1–T25 in Figure 2). The observation work was conducted at least twice at the same point under the same discharge conditions. The observation time period was set as 3–6 min. The observation frequency was set as 1000 Hz. Due to the daily, traffic and industrial noise in the county [26,27], especially with respect to the LFN generated by the surrounding living environments [8,28,29], the observation work was carried out in the evening to avoid signal mixing or distortion and ensure, as much as possible, the validity of the flow-induced LFN data. Table 1 shows the details of the observed discharge conditions of Xiangjiaba.

#### 2.1.3. Prototype Observation Data

**Figure 3:**
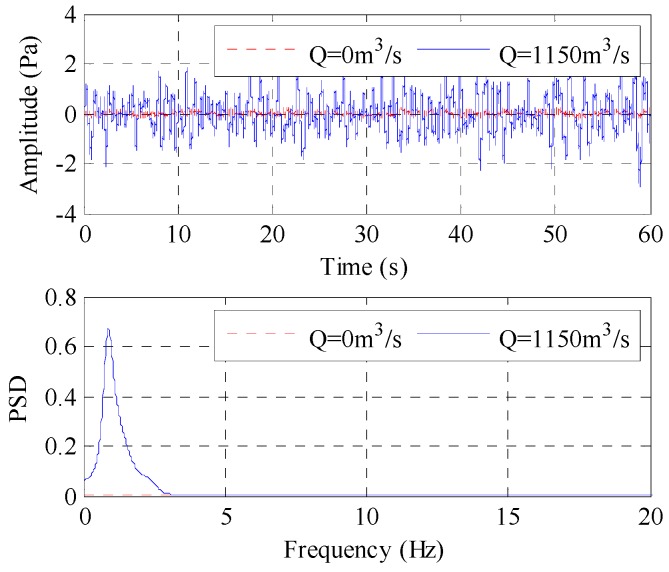
Comparisons of the time history and PSD curves of LFN.

Figure 3 compares the time history and power spectral density (PSD) curves of the LFN observed at T8 between the no-releasing discharge condition (Condition 1) and the small discharge condition (Condition 2) in Table 1. In this study, all of the PSD for the LFN is estimated using the autoregressive (AR) model with the Burg algorithm, which is one of the most frequently used parametric methods for processing various signals [30,31], and the order of the AR model is determined by the final prediction error (FPE) criterion. As shown, during the flood release period, the LFN intensity increases greatly, and the LFN energy presented in the PSD has an apparent peak frequency below 20 Hz. It is obvious that LFN is induced in the process of flood discharge.

##### Spatial Distribution and Propagation Patterns

**Figure 4:**
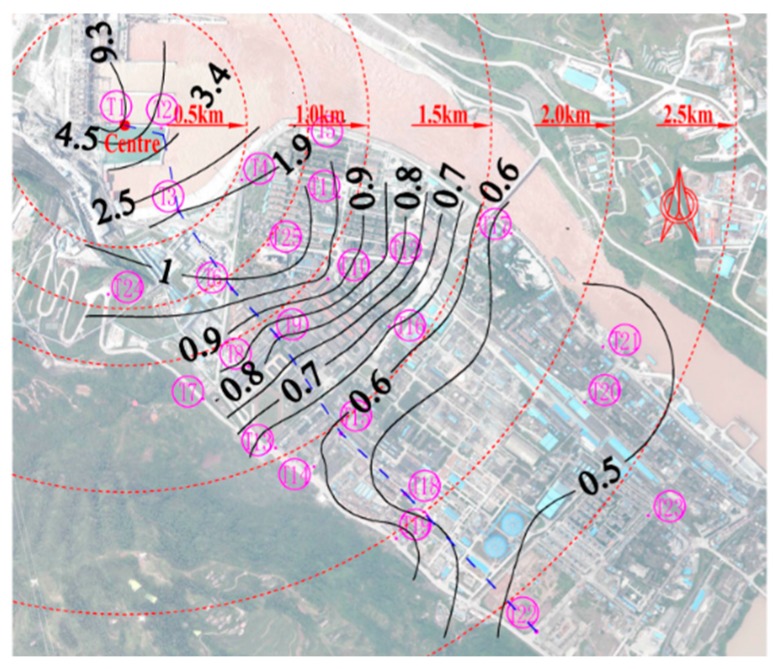
Distribution of the LFN amplitude. Q = 6600 m^3^/s; the unit is Pa.

The spatial distribution patterns of the induced LFN along the downstream area were obtained through statistical analysis. The LFN amplitude decreases gradually with increasing distance to the dam area under all observed discharge conditions. Figure 4 shows the contour map of the LFN amplitude observed under Condition 8. In this condition, the LFN amplitude achieved the maximum value, approximately 9.3 Pa (113.3 dB), at the dam area. To evaluate the human hearing and perception sensitivity for the LFN, the G-weighting (dB(G)) [32] is proposed as an appropriate metric for noise limits for LFN [33,34]. The maximum G-weighting SPL for the observed LFN was calculated as approximately 78.6 dB(G), well below the average 95 dB(G) hearing threshold [35] and the LFN limit of 85 dB(G) from Australian and Danish recommendations [36,37]. Therefore, it is noted that, under the discharge conditions in Table 1, the LFN observed around Xiangjiaba has no direct negative effect on local residents’ health in general.

**Figure 5:**
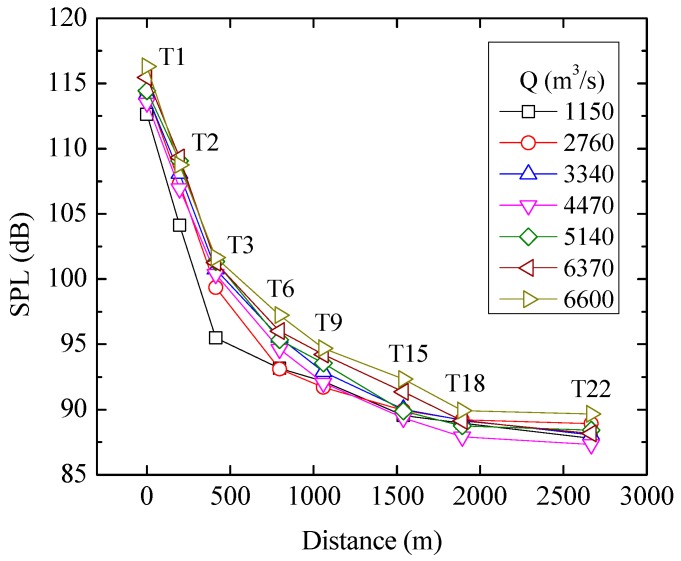
SPL distribution along the downstream city.

To analyze the attenuation rates of the flow-induced LFN in the process of propagation along the downstream city, the SPLs of 8 LFN observation points (dashed line in Figure 2) under all observed discharge conditions were calculated, as observed in Figure 5. Table 2 shows the SPL attenuation coefficients at different distances from the flood release and energy dissipation area. As a result, the SPLs near the dam area are greatly attenuated and have a maximum attenuation coefficient, 0.0347 dB/m, as a statistical average. The SPL attenuation coefficient gradually decreases with increasing distance to the dam area, and it decreases to 0.000459 dB/m for distances greater than 2 km.

##### Correlation between LFN and Discharge Flow Regime

**Figure 6:**
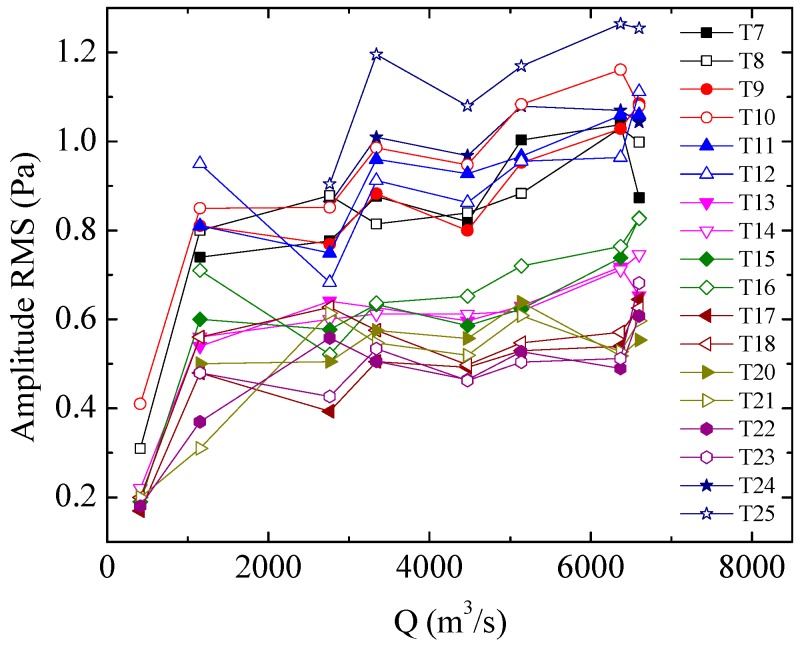
Correlation between the LFN intensity and Q.

**Figure 7:**
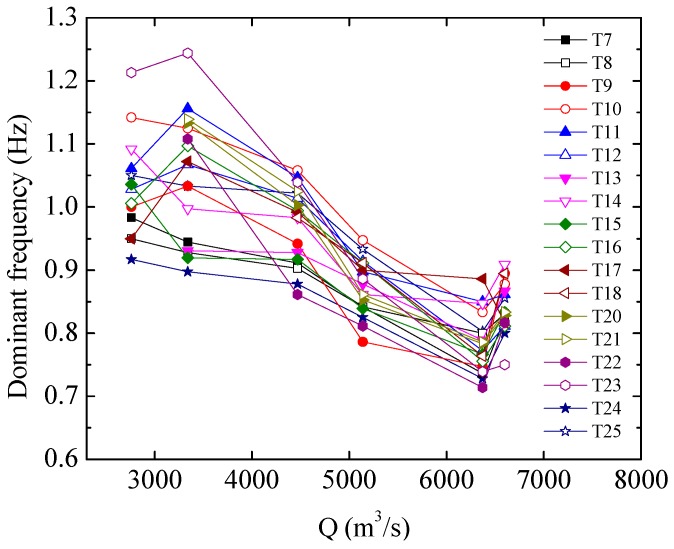
Correlation between the LFN dominant frequency and Q.

Figure 6 shows the relationship between the RMS values of the LFN amplitude analyzed and the flow rate for several observation points in the downstream city area. In general, the LFN amplitude increases gradually with increasing Q, and the discharge operation conditions have some influence on the LFN amplitude. It is seen from Table 1 and Figure 6 that the joint discharge operation schemes of the crest overflowing orifices and mid-discharge orifices are advantageous for reducing the LFN amplitude. For instance, the LFN observed under Condition 4 (Q = 3340 m^3^/s) has a higher amplitude than the LFN observed under Condition 5 (Q = 4470 m^3^/s). The LFN amplitude observed under Condition 8 (Q = 6600 m^3^/s) shows a significant decrease compared to the amplitude observed under Condition 7 (Q = 6370 m^3^/s). As joint discharge operation schemes can avoid concentrated flow in the stilling basin, maintaining uniform and smooth flow in the stilling basin plays a significant role in reducing the LFN amplitude induced by flood discharge. Figure 7 shows the relationship between the analyzed dominant frequency of the LFN and the flow rate. As shown in the figure, the dominant frequencies of the LFN are concentrated between 0 Hz to 2 Hz and decrease gradually with increasing Q under the observed discharge conditions.

### 2.2. Theoretical Model of Vortex Sound

In acoustic research, Powell [38] and Howe [39,40] *et al*. studied the basic issues of the internal mechanism of fluid vocalization and the interaction between acoustic wave and turbulent flow in terms of vortex dynamic theory since the 1960s, and they established the vortex sound theory. Their research results indicated that the vortex is a significant acoustic source and that vortex sound theory is feasible and effective for identifying the generation mechanism of flow-induced noise at a low Mach number. In the turbulent flow field of flood discharge and energy dissipation from a high dam, various vortices always exist. In a stilling basin, the large amount of energy carried by high-velocity submerged jets is dissipated through strong turbulent mixing. Numerous low-frequency large coherent structures are present in the strong shear layers of the submerged jets, and they have evident vortical structures, high regularity and repeatability. The acoustic energy’s formation and transition coming from the interaction of the vortical structures, potential flow and solid boundaries cannot be neglected. Therefore, the vortex sound theory is applied to determine the mechanism for LFN induced by energy dissipation through submerged jets in this study. Assuming that the flow is incompressible and isentropic and based on the equations of continuity and motion, the vortex sound equation [38] can be deduced and simplified as:

(1) $$\nabla^{2}p-\frac{1}{c^{2}}\frac{\partial^{2}p}{\partial t^{2}}=-\nabla \cdot \rho \left(\omega \times u\right),$$

where *p* is the acoustic pressure in the far field; *c* is the acoustic velocity; *ρ* is the density; ***ω*** is the vorticity vector; and ***u*** is the flow velocity vector. The equation shows that the vortex sound equation is a typical nonhomogeneous wave equation, with the differential expression of the acoustic wave’s propagation process in a nonhomogeneous fluid on the left and the acoustic source term of the vortex on the right. Equation (1) indicates that the flow-induced acoustic pressure is directly related to the sizes, variations and motion of the vortices. To predict the acoustic pressure, the general solution of Equation (1) for the far field is written as follows using the Green function method and the Helmholtz vortex equation, according to Möhring [41]:

(2) $$p(x,t)=\rho \frac{\partial }{\partial t^{\prime }}\int G\left(x,t;y,t^{\prime }\right)\cdot \omega \left(y,t^{\prime }\right)d^{3}ydt^{\prime },$$

where ***x*** is the displacement vector of the predicted point in the far field and ***y*** is the displacement vector of the acoustic source point in the flow region. The Green function must satisfy $\nabla_{y}G\left(x,t;y,t^{\prime }\right)=\nabla_{y}\times G\left(x,t;y,t^{\prime }\right)$.

The integrand on the right side of Equation (2) only contains the vorticity variable ***ω***, which identifies that the region with time-varying vorticity is the effective region of the acoustic source. Furthermore, because the flow velocity is not contained in the integrand, the acoustic pressure of the far field can be directly calculated by the effective vorticity fluctuation data. As ***G*** is a symmetric function, according to Powell’s deduction, ***G*** can be confirmed as:

(3) $$G\left(x,t;y,t^{\prime }\right)=\frac{1}{12\pi c^{2}x^{3}}\delta^{\prime \prime }\left(t-t^{\prime }-\frac{x}{c}\right)\left(x\cdot y\right)x\times y,$$

where *δ* is the Dirac delta function. Then, Equation (2) leads to:

(4) $$p(x,t)=\frac{\rho }{12\pi c^{2}x^{3}}\frac{\partial^{3}}{\partial t^{3}}\int \left(x\cdot y\right)x\times y\cdot \omega d^{3}y,$$

where the vorticity values depend on the time *t*′ = *t* − *x*/*c*. As the attenuation of acoustic pressure during propagation is not included above, a sonar equation is obtained as:

(5) $$SL-TL=20\log_{10}\frac{P_{e}}{P_{ref}}-{\left(\int_{L}\alpha dL\right)}_{y}=20\log_{10}\frac{P^{\prime }}{P_{ref}},$$

where *SL* is the RMS value of the SPL without regard to attenuation; *TL* is the SPL attenuation value; *P_e_* is the RMS value of acoustic pressure calculated by Equation (4); *P_ref_* is the reference value of the acoustic pressure, set as 2 × 10^−5^ Pa; *α* is the SPL attenuation coefficient; and *P′* is the virtual value of the acoustic pressure finally achieved with regard to attenuation. The mathematical prediction model of LFN is generated using Equations (4) and (5), and the LFN intensity can be predicted by substituting the results of numerical simulation of the near-field turbulent flow into the prediction model.

### 2.3. Numerical Turbulent Flow Model

#### 2.3.1. Gas-Liquid Turbulent Flow Model

A large number of numerical simulation studies have shown that the *k-ε* turbulent flow model is a reasonable method for simulating the hydrodynamic characteristics of turbulent flow [42,43]. However, the RNG *k-ε* turbulent flow model, first developed by Yakhot and Orszag [44], can better simulate flow with high strain rate and large streamline curvature compared to the traditional *k-ε* turbulent flow model. The VOF method, proposed by Hirt and Nichols [45] in 1975 based on the MAC method, is also an effective way to calculate the complex free water surface [46]. Therefore, the RNG *k-ε* turbulent flow model and the VOF method are employed in this study to analyze the hydraulic parameters in the flow field of flood discharge and energy dissipation of a high dam. The major governing equations are listed below:

Basic equations of turbulent flow:

(6) $$\left\{ \begin{array}{l} \frac{\partial \rho }{\partial t}+\frac{\partial \rho u_{i}}{\partial x_{i}}=0\\ \frac{\partial u_{i}}{\partial t}+\frac{\partial u_{i}u_{j}}{\partial x_{j}}=-\frac{1}{\rho }\frac{\partial p}{\partial x_{i}}+\frac{1}{\rho }\frac{\partial }{\partial x_{j}}\left[\left(\mu +C_{\mu }\frac{k^{2}}{\varepsilon }\right)\left(\frac{\partial u_{i}}{\partial x_{j}}+\frac{\partial u_{j}}{\partial x_{i}}\right)\right] \end{array}\right.,$$

*k* equation:

(7) $$\frac{\partial \left(\rho k\right)}{\partial t}+\frac{\partial \left(\rho ku_{i}\right)}{\partial x_{i}}=\frac{\partial }{\partial x_{j}}\left[\alpha_{k}\mu_{eff}\frac{\partial k}{\partial x_{j}}\right]+G_{k}+\rho \varepsilon ,$$

*ε* equation:

(8) $$\frac{\partial \left(\rho \varepsilon \right)}{\partial t}+\frac{\partial \left(\rho \varepsilon u_{i}\right)}{\partial x_{i}}=\frac{\partial }{\partial x_{j}}\left[\alpha_{\varepsilon }\mu_{eff}\frac{\partial \varepsilon }{\partial x_{j}}\right]+\frac{C_{1\varepsilon }^{*}\varepsilon }{k}G_{k}-C_{2\varepsilon }\rho \frac{\varepsilon^{2}}{k},$$

Gas-liquid VOF equation:

(9) $$\left\{ \begin{array}{l} \alpha_{a}=1-\alpha_{w}\\ \frac{\partial \alpha_{w}}{\partial t}+u_{i}\frac{\partial \alpha_{w}}{\partial x_{i}}=0 \end{array}\right.,$$

where $\mu_{eff}=\mu +\rho C_{\mu }\frac{k^{2}}{\varepsilon }$, $C_{1\varepsilon }^{*}=C_{1\varepsilon }-\frac{\eta \left(1-\eta /\eta_{0}\right)}{1+\beta \eta^{3}}$, $\eta ={\left(2E_{ij}\cdot E_{ij}\right)}^{1/2}\frac{k}{\varepsilon }$, $E_{ij}=\frac{1}{2}\left(\frac{\partial u_{i}}{\partial x_{j}}+\frac{\partial u_{j}}{\partial x_{i}}\right)$, *C_μ_* = 0.0845, *α_k_* = *α_ε_* = 1.39, *C*_1_*_ε_* = 1.42, *C*_2_*_ε_* = 1.68, *η*_0_ = 4.377, *β* = 0.012, and *α_w_* and *a_a_* are the volume fractions of water and air in a unit grid, respectively.

#### 2.3.2. Simulation Domain and Boundary Conditions

**Figure 8:**
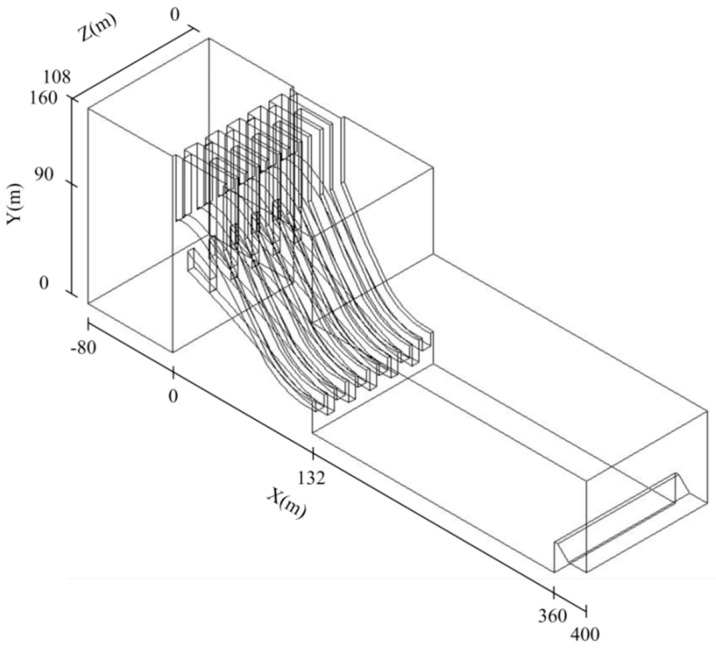
Turbulent flow model and simulation domain.

The numerical turbulent flow model established is for a 1:1 scale simplified spillway dam of Xiangjiaba, as shown in Figure 8, where X is along the flow direction. The calculation conditions of the numerical simulation are listed in Table 1. The simulation domain includes the water body extended 80-m into the reservoir; the spillway dam segments with the crest overflowing orifices and mid-discharge orifices, which are 132 m long; the 228 m long stilling basin; and the 40 m long tail-weir. Because the stilling basins of Xiangjiaba are symmetrical on two sides of the middle guide wall, the simulations are done on just a half portion of the discharge structures which is on one side of the middle guide wall in the transverse Z direction to reduce the numeration workload. The model is meshed by block-structured grids. For the main flood discharge and energy dissipation regions, such as the spillway dam area and the front of the stilling basins, the grid size is set as 1 m long × 1 m wide × 1 m high. In the remaining regions, the grid size is set as 2 m long × 1 m wide × 1 m high. Mesh refinement is applied on the overflow dam face, and the mesh refinement method adaptive to the variations of the pressure gradient and free water surface is employed during the calculation. The total number of grid units is approximately 3.52 million. Furthermore, based on the RNG *k-ε* turbulent flow model and the VOF method, the methods of finite volume, PISO and wall function are employed in the process of calculation.

When setting the boundary conditions of the turbulent flow model, the upstream inlet and downstream outlet are set as the pressure inlet and pressure outlet, respectively, and the water levels of upstream and downstream are set equal to the measured values shown in Table 1. The top boundary of the model is all set as the air pressure inlet with the atmospheric pressure. The other boundary is set as a no-slip wall.

## 3. Results and Discussion

From solving Powell’s equation in the above section, it is suggested that the region with the time-varying vorticity is the effective region of acoustic source, and the mathematical prediction model of LFN is developed accordingly. The numerical turbulent flow model is also built as a feed to the LFN prediction model. In this section, the numerical calculation results from the turbulent flow model and the vortex sound model are analyzed to verify the theoretical analysis results. The flow field and vorticity fluctuation characteristics of the energy dissipation area are analyzed to identify the main regions of acoustic source for the LFN. The prediction model developed is used to calculate the LFN intensity on site.

### 3.1. Verification and Validation of Turbulent Flow Model

To evaluate the numerical uncertainty and discretization errors of the simulation results, the Grid Convergence Index (GCI) analysis based on the Richardson extrapolation method, which was outlined by Celik *et al*. [47,48], is adopted herein. Two additional refined grids are set to do the computations, which represent a relevant contribution to the numerical uncertainty. The three different grids consist of 3.52 (*N*_1_), 4.81 (*N*_2_) and 6.41 (*N*_3_) million cells respectively, which are called as the coarse, medium and fine grids. The GCI of the coarse and fine grids are calculated as follows:

(10) $$\left\{ \begin{array}{l} GCI_{coarse}^{21}=\frac{1.25e_{21}r_{21}^{P}}{r_{21}^{P}-1}\\ GCI_{fine}^{32}=\frac{1.25e_{32}}{r_{32}^{P}-1} \end{array}\right.,$$

where $P=\frac{1}{\ln\left(r_{21}\right)}\left|\ln\left|\left(f_{3}-f_{2}\right)/\left(f_{3}-f_{2}\right)\right|+q\left(P\right)\right|$, $e_{21}=\left|\frac{f_{1}-f_{2}}{f_{1}}\right|$, and $e_{32}=\left|\frac{f_{2}-f_{3}}{f_{2}}\right|$. Here *P* is the order of accuracy; *e* is the relative error; *r* is the grid refinement factor; and *f*_1_, *f*_2_ and *f*_3_ denote the solutions at the coarse, medium, and fine levels, respectively. As Roach [49] recommended a minimum 10% change in point of the computational cost and time consumption, the grid refinement factor is set to *r* = 1.11. The variables, *f*_1_, *f*_2_ and *f*_3_, are considered as the flow velocity magnitudes near the stilling basin floor for different grid sizes. 

Table 3 summarizes the numerical uncertainty assessment results for the turbulent flow model under Condition 8. All of the solutions for the velocity magnitudes show good mesh convergence behavior with errors of less than 2.5%, even to the coarse grid.

**Figure 9:**
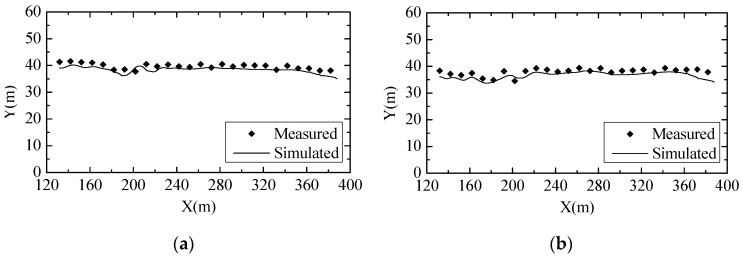
Comparisons of the water surface profile in the stilling basin between the measured (from the physical model) and simulated results: (**a**) Midline of the crest overflowing orifice; (**b**) Midline of the mid-discharge orifice.

**Figure 10:**
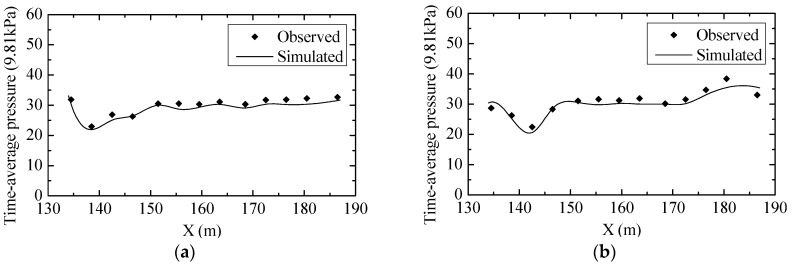
Comparisons of the time-average pressure distribution on the stilling basin floor between the observed (from the prototype) and simulated results: (**a**) Midline of the crest overflowing orifice; (**b**) Midline of the mid-discharge orifice.

**Figure 11:**
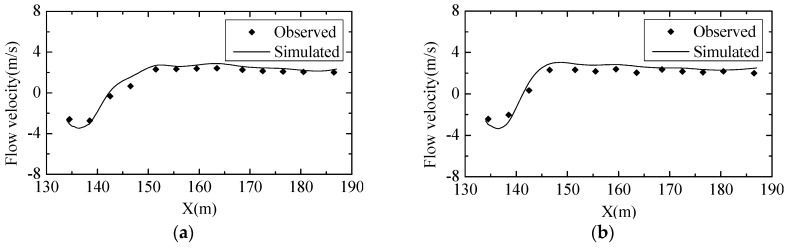
Comparisons of the flow velocity near the stilling basin floor between the observed and simulated results: (**a**) Midline of the crest overflowing orifice; (**b**) Midline of the mid-discharge orifice.

To further validate the turbulent numerical simulation model, the data among the physical model experiment, hydraulic prototype observation and numerical simulation results were compared. The measured and simulated results of the water surface profile of Condition 8 in the stilling basin are shown in Figure 9. The observed and simulated results of the time-average pressure distribution and flow velocity near the stilling basin floor of Condition 8 are shown in Figure 10 and Figure 11, respectively. 

The figures show that the simulation results of the water surface profile, pressure distribution and flow velocity near the floor are in good agreement with those from the experiments and observations, and the other conditions’ validation results are all consistent. As a result, it is feasible to analyze the hydraulic characteristics of the flow field by the numerical methods chosen in this study.

### 3.2. Analyses of Numerical Modeling Results

#### 3.2.1. Flow Velocity Distribution

**Figure 12:**
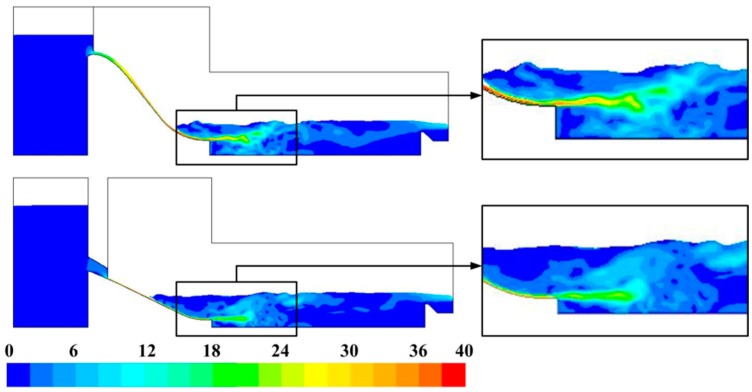
Flow velocity contours of the midline sections of the crest overflowing orifice and mid-discharge orifice. The unit is m/s.

Because the distribution patterns of the flow field obtained by numerical calculation under every condition are similar, only the velocity contours on the sections along midlines of the crest overflowing orifice and mid-discharge orifice of Condition 8 are shown here in Figure 12. In Figure 12, the flow velocity begins to increase after the water discharges from the reservoir through the orifices, and the maximum velocity reaches 39.67 m/s near the end of the overflow dam surface. Then, the high velocity flow enters the stilling basin and forms submerged jets, where the water level rises locally. The multi-horizontal submerged jets maintain a certain distance in space from each other, and a steady three-dimensional flow structure similar to a submerged hydraulic jump is developed with strong turbulence and shearing action. As a result, the enormous energy carried by the high velocity flow is dissipated. Nevertheless, the kinetic energy of the high-velocity jets cannot vanish until the jets nearly reach the middle of the stilling basin. 

**Figure 13:**
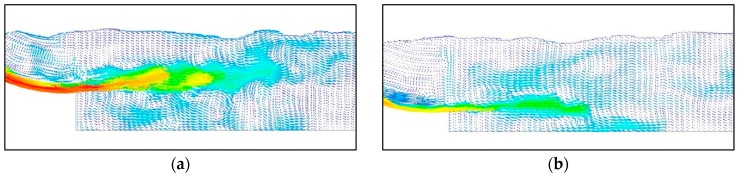
Flow velocity vector distribution in the energy dissipation area: (**a**) Midline of the crest overflowing orifice; (**b**) Midline of the mid-discharge orifice.

Figure 13 shows the instantaneous velocity vector distribution. Plenty of vortices of different sizes and strengths form and develop continuously around the high-velocity submerged jets in the zone of the three-dimensional flow structure (submerged hydraulic jump) as a result of the large flow velocity gradient.

#### 3.2.2. Vortical Structures’ Characteristics

When analyzing the characteristics of the vorticity field, the Eulerian method, such as the Q-criterion developed by Hunt *et al*. [50], is frequently utilized to identify and visualize the vortical structures, and the patterns of the vortex motion can be found in an instantaneous flow field [51]. In addition, the Q-criterion method relies on the derivatives of the velocity and is derived from the invariants of the velocity gradient tensor. The velocity gradient tensor *A* for incompressible flow can be divided into two parts, *S* and *W*, respectively, for the symmetric and antisymmetric parts:

(11) $$A_{ij}=S_{ij}+W_{ij}=\frac{1}{2}\left(\frac{\partial u_{i}}{\partial x_{j}}+\frac{\partial u_{j}}{\partial x_{i}}\right)+\frac{1}{2}\left(\frac{\partial u_{j}}{\partial x_{i}}-\frac{\partial u_{i}}{\partial x_{j}}\right),$$

where *S_ij_* is the strain rate tensor representing the irrotational motion, and *W_ij_* is the rotation tensor representing the rotational motion. *Q* is the second invariant of A_ij_ defined as:

(12) $$Q=\frac{1}{2}\left(W_{ij}W_{ij}-S_{ij}S_{ij}\right),$$

**Figure 14:**
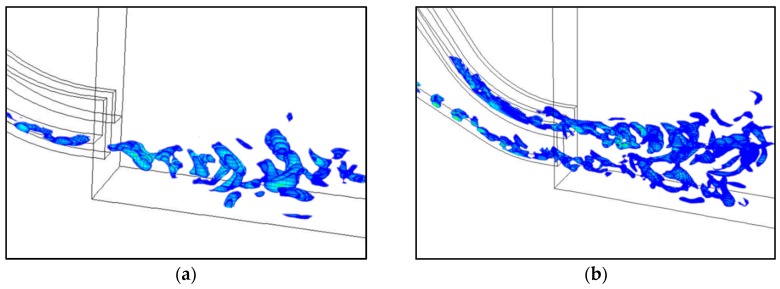
Instantaneous vortical structures in the energy dissipation area by *Q* = 10: (**a**) Condition 2; (**b**) Condition 8.

**Figure 15:**
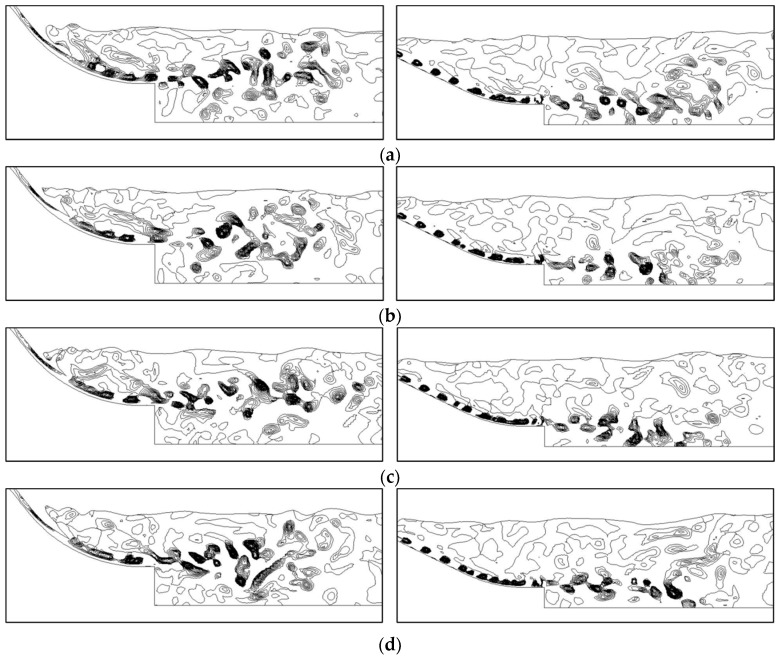
Contours of the *Q* value (*Q* > 0) of the midline sections of the crest overflowing orifice and mid-discharge orifice in incremental time steps: (**a**) t_0_ = 180 s; (**b**) t_0_ + 1 s; (**c**) t_0_ + 2 s; (**d**) t_0_ + 3 s.

As a consequence, if the *Q* value in some regions is positive, the rotational motion plays a dominating role in those regions. Figure 14 displays the visualization results of the vortical structures in the flow field of energy dissipation by the Q-criterion. To display the interaction of the vortices more clearly in the process of energy dissipation, the contours of the *Q* value (*Q* > 0) of the midline sections of the crest overflowing orifice and mid-discharge orifice under Condition 8 are presented in incremental time steps in Figure 15. 

At different discharge moments, the shear layers grow gradually along the overflow dam surface with increasing flow velocity, where the vortical structures that are tubular in the horizontal axial direction (Z direction) begin to appear. At the end of the overflow dam surface and the beginning of the submerged jets, the vortical structures in the shear layers turn to axial breakup.The breakup becomes more evident when the submerged jets enter the stilling basin, and much more vortical structures of different sizes and strengths are developed. It is seen that the three-dimensional characteristics of the flow regime in the stilling basin are distinct, and the vortical structures in the flow field are unsteady, movable and intermittent under different discharge conditions. All of the visualization results show that the vortical structures are mainly located at the beginning of the stilling basin, where the shearing motion of the flow is violent.

#### 3.2.3. Correlation Analysis of Vorticity Fluctuating Characteristics and Acoustic Source

**Figure 16:**
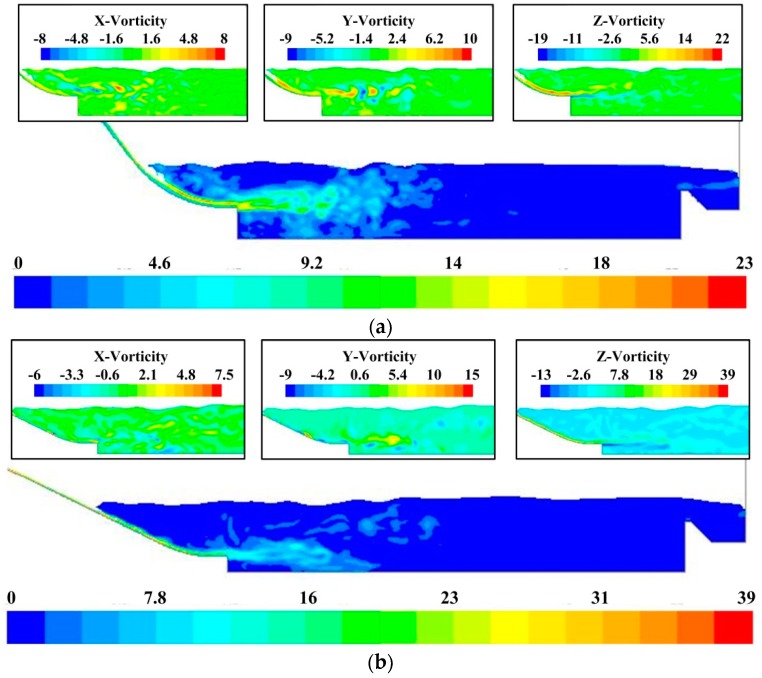
Vorticity contours in the energy dissipation area: (**a**) Midline of the crest overflowing orifice; (**b**) Midline of the mid-discharge orifice. The unit is s^−1^.

As the radiated acoustic pressure in the far field is directly related to the vorticity fluctuation characteristics and vortex motions in the flow region according to previous theoretical deductions, the dynamic characteristics of the vorticity in the energy dissipation area are derived by numerical calculation in this section to verify the deductions and estimate the location of the effective regions of acoustic source for the LFN. Figure 16 shows the vorticity contours of the midline sections of the crest overflowing orifice and mid-discharge orifice of Condition 8. It is clearly observed that the distributions of the vorticity and velocity fields are approximately similar. The vorticity intensity is large in two regions, one located at the free surface of the overflow water, and the other one located at the strong shear layers around the submerged jets. Before the jets get into the stilling basin, the Z-component of the vorticity, representing the quantity of horizontal vortices in the shear layers, is maximum. When the horizontal vortices flow into the stilling basin, vertical vortices begin to develop, and the components of the vorticity in every direction tend toward uniformity. The distributions of the vorticity field correspond to the identification results of the vortical structures in Figure 14 and Figure 15.

**Figure 17:**
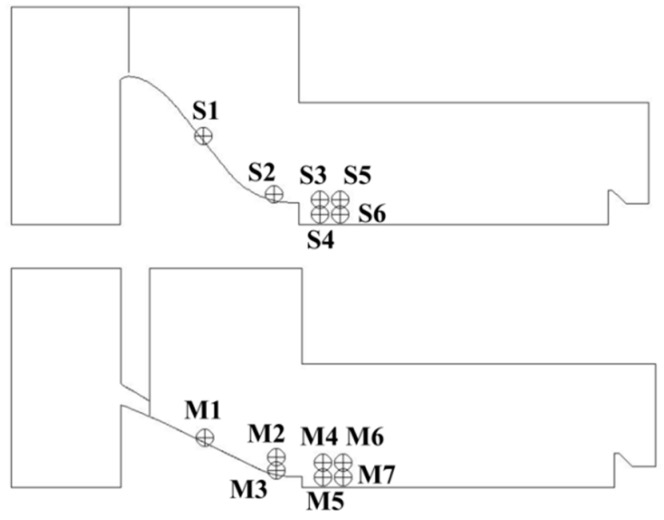
Vorticity monitoring points on the midline sections of the crest overflowing orifice and mid-discharge orifice.

To analyze the vorticity fluctuation characteristics in the flow field, several monitoring points have been set on the midline sections of the crest overflowing orifice and mid-discharge orifice in the numerical models, as shown in Figure 17. Specifically, S1 and M1 are set at the free surface of the overflow water; S2 and M2 are set under the high-velocity overflow water; and the rest of the measuring points are set in the energy dissipation region of the submerged jets.

The time-averaged values, RMS values and dominant frequencies of the vorticity fluctuation of every monitoring point under all conditions were computed according to the transient monitoring data obtained through the numerical calculation. The results for Condition 8 are listed in Table 4. It is shown that, for the monitoring points located at the free surface of overflow water (S1, M1), the time-averaged values of the vorticity fluctuation are larger, whereas the RMS values are smaller, and the dominant frequency does not exist. The points located at the strong shear layers around the high-velocity submerged jets (S4, S5, S7, M4, M6, and M8) have the vorticity fluctuation with larger RMS values and dominant frequencies of 0–2 Hz.

**Figure 18:**
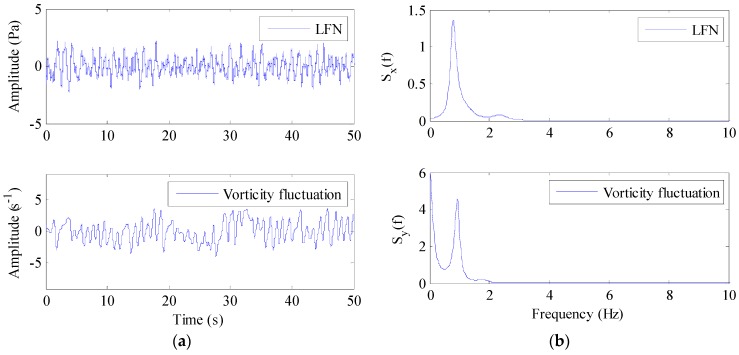
Comparisons between the vorticity monitoring point S5 and the prototype observation point T8: (**a**) Time-history curve; (**b**) Autocorrelation PSD.

Contrastive analyses were conducted between the monitoring data of the vorticity fluctuation and the prototype observation data of the LFN under the same condition. Figure 18 compares the time-history curves and autocorrelation PSD between monitoring point S5 and prototype observation point T8 under Condition 8. As a consequence, the vorticity fluctuation data from the strong shear layers (S4, S5, S7, M4, M6, and M8) have approximately the same periodic pattern with the prototype observation data of LFN.

**Figure 19:**
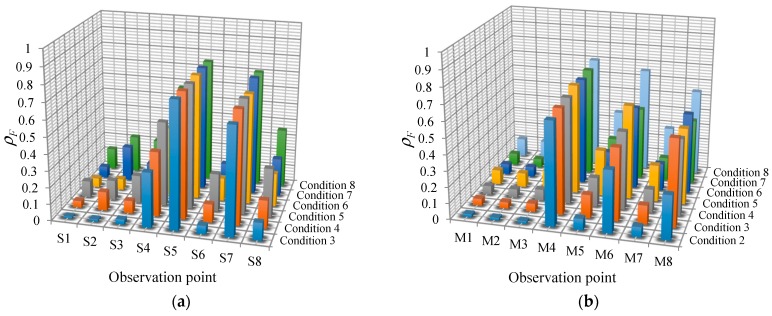
Statistical distribution of the spectral correlation coefficients between the vorticity monitoring points and the prototype observation point: (**a**) Midline of the crest overflowing orifice; (**b**) Midline of the mid-discharge orifice.

Additionally, for clearly quantifying the correlation between the vorticity fluctuation and LFN, the spectral correlation coefficient *ρ_F_* [52] is defined as:

(13) $$\rho_{F}=\frac{\sum_{k=1}^{n}F_{x}\left(k\right)F_{y}\left(k\right)}{\sqrt{\sum_{k=1}^{n}F_{x}^{2}\left(k\right)\sum_{k=1}^{n}F_{y}^{2}\left(k\right)}},$$

where *F_x_* and *F_y_* represent the module of the Fourier spectrum of the vorticity fluctuation and the observed LFN, respectively. Figure 19 displays the statistical results of the spectral correlation coefficients between every monitoring point of the vorticity fluctuation and the prototype observation point T8 under all conditions. It is seen that the values of *ρ_F_* at the monitoring points in the strong shear layers (S4, S5, S7, M4, M6, and M8) are relatively larger, which can be up to 0.762. The average values of *ρ_F_* of the monitoring points in the strong shear layers of the crest overflowing orifice and the mid-discharge orifice are 0.681 and 0.601, respectively.

The analysis results of the vorticity fluctuating characteristics above verify the close correlation between the vorticity fluctuation in the strong shear layers around the high-velocity submerged jets and the flow-induced LFN propagated to the far field. In addition, combined with the identification results of the vortical structures above, it is concluded that, during the energy dissipation process of the submerged jets, a large number of turbulent low-frequency large-scale vortical structures come into existence in the strong shear layers around the submerged jets because of the large velocity gradient, and the LFN is generated in the intensive interactions of the vortical structures. Therefore, the strong shear layers around the submerged jets are judged as the main regions of acoustic source for the LFN.

### 3.3. Verification of the Mathematical Prediction Model of LFN

According to the mathematical prediction model of LFN (Equations (4) and (5)) built theoretically in the previous section, the numerical simulation results of the main acoustic source regions in the flow field are put into the prediction model to calculate the LFN intensity of prototype observation point T1, which is located at the energy dissipation region under all conditions. The comparisons between the prediction results and prototype observation results are listed in Table 5.

**Figure 20:**
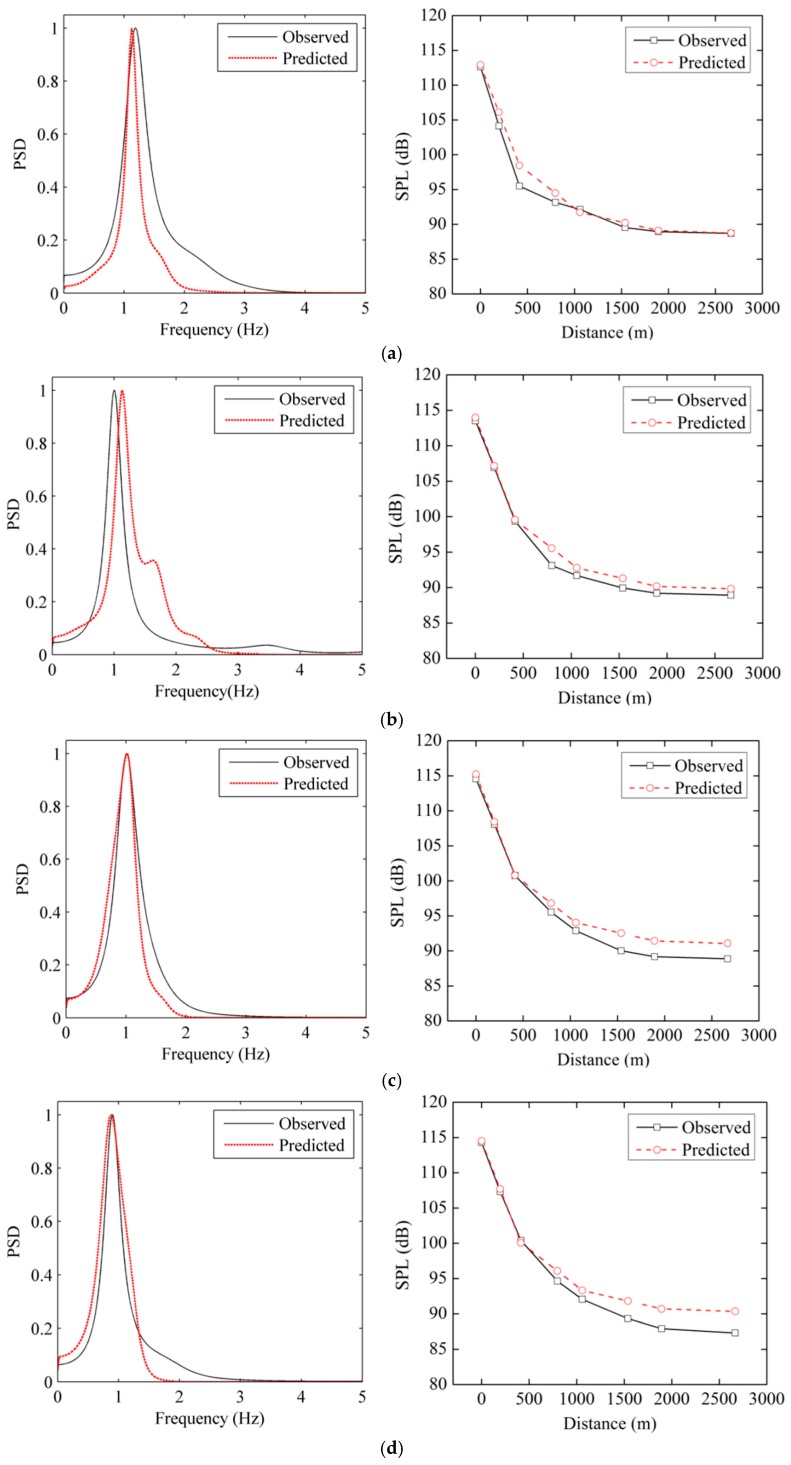

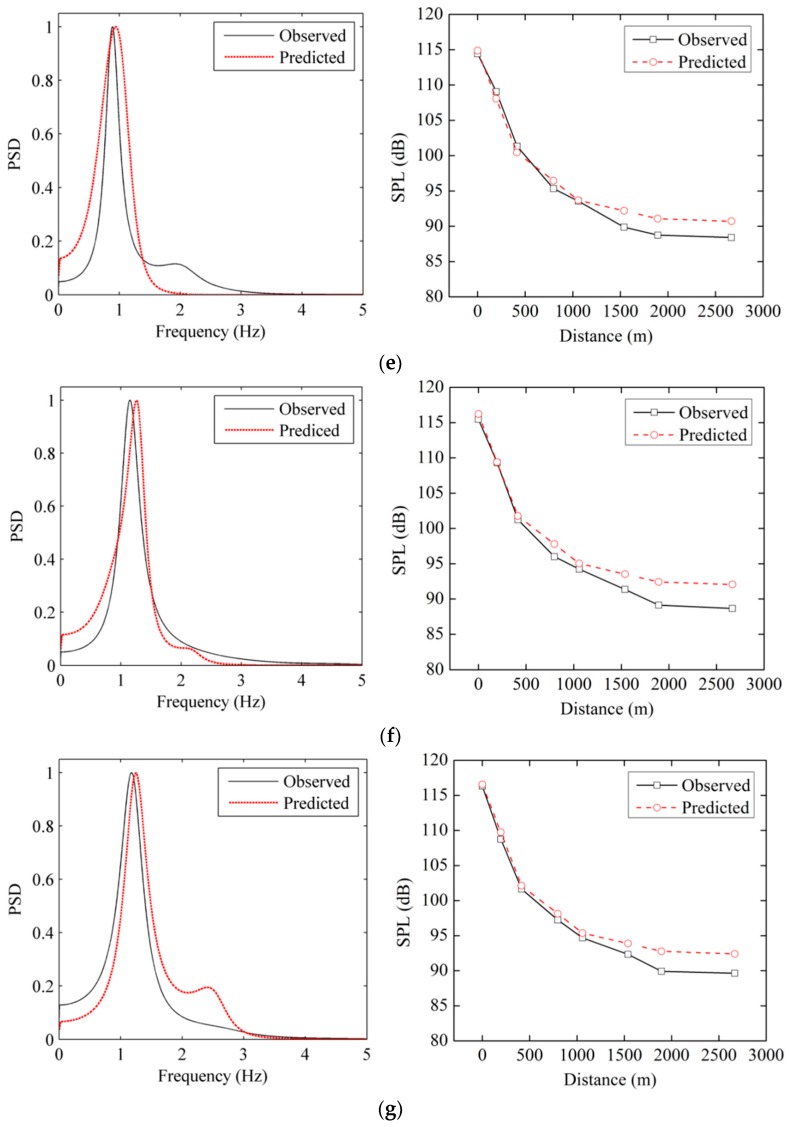
Comparisons of the normalized PSD of T1 and the LFN amplitudes along the downstream between the observed and predicted data: (**a**) Condition 2; (**b**) Condition 3; (**c**) Condition 4; (**d**) Condition 5; (**e**) Condition 6; (**f**) Condition 7; (**g**) Condition 8.

The comparison results indicate that the LFN amplitudes, dominant frequencies and variation laws predicted theoretically are approximately consistent with those of the prototype observation. The predicted amplitudes gradually increase with increasing flow rate, and the dominant frequencies of the LFN predicted are concentrated between 0 Hz and 2 Hz. Depending on the prediction results of T1 and the SPL attenuation rate in Table 2, the LFN amplitudes of several observation points (T2, T3, T6, T9, T15, T18, and T22) downstream were estimated. Figure 20 compares the normalized PSD of T1 and the LFN amplitudes along the downstream between the observed and predicted LFN data under all conditions. It is shown that the variation tendencies of the LFN amplitudes of the observed and predicted LFN are nearly the same, while the predicted LFN amplitudes of some points are a little larger than the observed data. 

**Figure 21:**
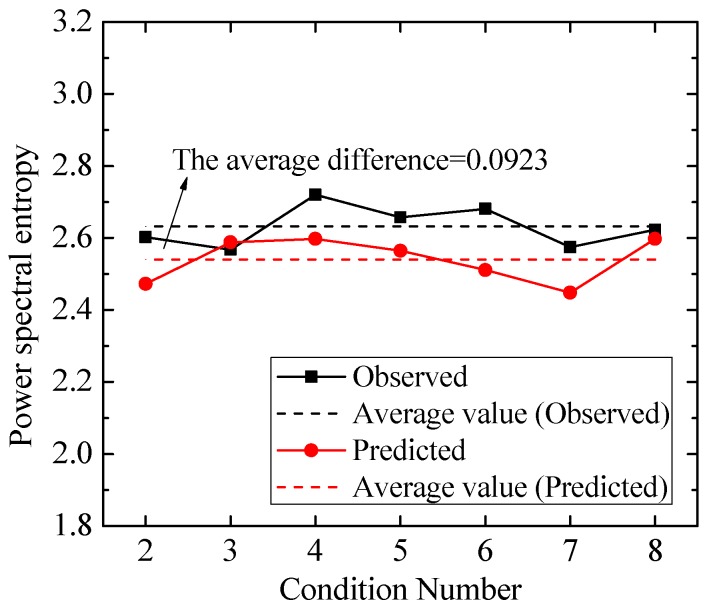
Comparisons of the power spectral entropy between the observed and predicted LFN data.

In order to evaluate the proposed prediction model, the power spectral entropy, derived from Shannon entropy [53] as a common method [54,55], is introduced here to quantify and compare the uncertainties and complexities of PSD estimation between the observed and predicted LFN data. The power spectral entropy is defined as [54,55]:

(14) $$H_{F}=-\sum_{f=0.1}^{f=20}p\left(f\right)\log\left(p\left(f\right)\right),$$

where $p\left(f\right)=s\left(f\right)/\sum_{f=0.1}^{f=20}s\left(f\right)$, *s*(*f*) is the spectral component, the base of the logarithm is two, and the unit of *H_F_* is bits. In current work, because the infrasound microphone’ minimum measuring threshold is 0.1 Hz and the on-site LFN energy is generally below 20 Hz, the power spectral entropy is estimated based on the PSD within 0.1–20 Hz in Equation (14). In essence, the greater the spectral entropy, the more irregular the PSD distribution. The power spectral entropy between the observed and predicted LFN data of T1 under all conditions are shown in Figure 21. It is seen that the power spectral entropy of the observed and predicted LFN data are very close, and the power spectral entropy of the observed LFN are always a little larger than those of the predicted LFN. The average power spectral entropy of the observed and predicted LFN data are 2.632 and 2.540, respectively, and the difference in the average values is approximately 0.0923. A comparison of the results reveals that the PSD between the observed and predicted LFN data has uncertainties and complexities of nearly the same degree.

**Figure 22:**
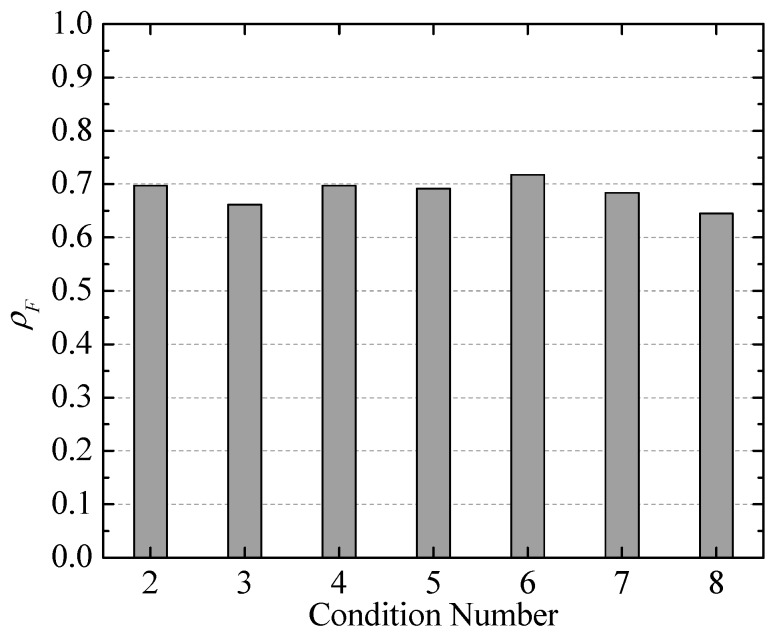
Spectral correlation coefficients between the observed and predicted LFN data.

Moreover, the spectral correlation coefficients *ρ_F_* between the observed and predicted LFN data of T1 were calculated through Equation (13). As observed in Figure 22, the values of *ρ_F_* under all conditions are relatively large, at approximately 0.645–0.717. Thus, according to the analyses of the uncertainties of PSD and the spectral correlation coefficients, it can be seen that the mathematical prediction model is feasible and reasonable for preliminary estimation of the LFN intensity induced by energy dissipation through the submerged jets of a high dam.

## 4. Conclusions

In this study, extensive prototype observations and analyses of low frequency noise (LFN) induced by the process of energy dissipation through the submerged jets of a high dam are carried out. It is found that the LFN amplitude reaches the maximum value near the dam area, which is below the hearing threshold and the LFN limits. In general, the observed LFN has no directly negative effect on local residents’ health. The sound pressure level (SPL) attenuation coefficient of LFN decreases gradually with increasing distance to the dam area, reaching maximum in the vapor atomization area near the overflow dam. The flow-induced LFN intensity has a close relationship with the discharge operation schemes and flow regimes in the stilling basin, and maintaining uniform and smooth flow patterns in the stilling basin using the joint discharge operation scheme has the benefit of reducing the LFN intensity.

The results from numerical simulation of the flow field characteristics of energy dissipation by the submerged jets indicate that the vortical structures are mainly located at the beginning of the stilling basin where the shearing motion of the flow is violent. The results from correlation analysis of the vorticity fluctuation characteristics and acoustic source indicate that the vorticity fluctuation from the strong shear layers around the high-velocity submerged jets have larger RMS values, where the vorticity fluctuation data are highly correlated to the on-site LFN data. Besides, the strong shear layers are the main regions of acoustic source for the LFN. The mathematical prediction model of the LFN intensity for energy dissipation by the submerged jets is established by combining the vortex sound theory and turbulent flow model, and the model is verified by the prototype observations. The intensity and dominant frequency of the predicted and observed LFN data show satisfactory agreement. 

This study for the first time provides the reference data, theoretical foundation and prediction method for addressing the environmental problem of LFN induced by energy dissipating submerged jets during flood discharge from a high dam. In further studies, the specific and scientific discharge operation schemes for reducing the LFN intensity around the station should be built using the current models, which will provide an operation guide and improvements for the station in engineering practices. The theoretical and numerical methods adopted in this study should be applied to other similar engineering projects on LFN issues for further verification of the universality of the prediction model and optimization of the model parameters. Moreover, further problems, such as the propagation and attenuation patterns of LFN and the effects of the water surface wave on the LFN energy, should be studied systematically.

## Figures and Tables

**Table 1:** Observed discharge conditions *.

No.	Q (m^3^/s)	Water Level Elevation (m)		Crest Overflowing Orifice		Mid-Discharge Orifice	
		Upstream	Downstream	Number	Opening (m)	Number	Opening (m)
1	0	/	/	/	/	/	/
2	1150	353.43	267.71	/	/	M6, M8, M10	3.3
3	2760	379.42	269.81	C8–C11	1.5	M6–M10	1.5
4	3340	379.34	270.52	C8–C11	1.5	M1–M10	1.5
5	4470	379.42	271.27	C2–C5, C8–C11	1.5	M1–M10	1.5
6	5140	379.30	271.87	C2–C5, C8–C11	1.7	M1–M10	1.9
7	6370	379.19	272.77	C2–C5, C8–C11	3.0	M1–M10	2.0
8	6600	374.71	275.89	C1–C6	3.0	M1–M10	1.2
				C7–C12	2.0		

* Q is the total discharge through the dam orifices. The discharge orifice’s number is named from the left bank to the right bank.

**Table 2:** SPL attenuation coefficients at different propagation ranges *.

Range	Q (m^3^/s)							Statistical Average
	1150	2760	3340	4470	5140	6370	6600	
Dam area	0.0434	0.0336	0.0322	0.0356	0.0274	0.0314	0.0386	0.0347
<0.5 km	0.0251	0.0220	0.0213	0.0202	0.0224	0.0234	0.0206	0.0221
0.5–1 km	0.00594	0.0108	0.0118	0.0124	0.0112	0.0102	0.0106	0.0104
1–2 km	0.00236	0.00190	0.00276	0.00319	0.00360	0.00406	0.00388	0.00311
>2 km	0.000324	0.000368	0.000397	0.000777	0.000416	0.000596	0.000332	0.000459

* The unit is dB/m.

**Table 3:** Numerical uncertainty assessment for the turbulent flow model *.

*Z* (m)	*X* (m)	Velocity Magnitude (m/s)			*r*_21_	*r*_32_	*p*	*e*_21_	*e*_32_	$GCI_{coarse}^{21}$ (%)	$GCI_{fine}^{32}$ (%)
		*f*_1_	*f*_2_	*f*_3_							
44	155	2.628	2.610	2.599	1.11	1.11	4.28	0.0067	0.0043	2.33	0.96
	170	2.516	2.498	2.491	1.11	1.11	8.79	0.0072	0.0029	1.49	0.24
	185	2.182	2.171	2.176	1.11	1.11	7.73	0.0053	0.0024	1.19	0.24
54	155	2.773	2.763	2.768	1.11	1.11	5.79	0.0035	0.0019	0.95	0.29
	170	2.509	2.511	2.509	1.11	1.11	2.51	0.0009	0.0007	0.49	0.29
	185	2.411	2.400	2.395	1.11	1.11	8.56	0.0049	0.0020	1.04	0.18

* The solutions for the velocity magnitudes with Z = 44 m and Z = 54 m are at the midlines of the crest overflowing orifice and the mid-discharge orifice, respectively.

**Table 4:** Analyses of the vorticity monitoring data.

Midline Section of Crest Overflowing Orifice				Midline Section of Mid-Discharge Orifice			
Monitoring Point	Time-Averaged Value (s^−1^)	RMS Value (s^−1^)	Dominant Frequency (Hz)	Monitoring Point	Time-Averaged Value (s^−1^)	RMS Value (s^−1^)	Dominant Frequency (Hz)
S1	15.143	0.663	/	M1	30.021	1.656	/
S2	3.022	0.031	/	M2	7.986	0.394	/
S3	1.828	0.812	/	M3	0.654	0.243	/
**S4**	**18.737**	**1.149**	**1.000**	**M4**	**20.610**	**2.530**	**1.060**
**S5**	**11.686**	**1.599**	**0.960**	M5	1.630	0.665	/
S6	1.518	0.680	/	**M6**	**10.589**	**1.162**	**0.875**
**S7**	**8.948**	**1.839**	**1.020**	M7	1.791	0.967	/
S8	2.036	1.056	/	**M8**	**6.045**	**1.812**	**0.425**

**Table 5:** Comparisons of results from the prediction model and prototype observation of LFN *.

Condition	Prototype Observation Results			Prediction Model Results		
	LFN (Pa)	SPL (dB)	Frequency (Hz)	LFN (Pa)	SPL (dB)	Frequency (Hz)
2	8.57	112.64	1.117	8.84	112.91	1.150
3	9.49	113.53	1.022	9.99	113.97	1.125
4	10.72	114.59	0.978	11.54	115.23	0.933
5	10.38	114.30	0.899	10.62	114.50	0.895
6	10.54	114.44	0.833	11.08	114.87	0.975
7	11.87	115.47	0.894	12.93	116.21	1.261
8	13.08	116.31	1.178	13.46	116.56	1.250

* The values of the LFN intensity and SPL in the table are the RMS values.

## Abbreviations

The following abbreviations are used in this manuscript:

LFN	Low frequency noise
SPL	Sound pressure level
PSD	Power spectral density
AR model	Autoregressive model
FPE	Final prediction error
Q	Total discharge through the dam orifices (m^3^/s)
GCI	Grid Convergence Index
